# Breast Density and Breast Cancer Incidence in the Lebanese Population: Results from a Retrospective Multicenter Study

**DOI:** 10.1155/2017/7594953

**Published:** 2017-07-02

**Authors:** Christine Salem, David Atallah, Joelle Safi, Georges Chahine, Antoine Haddad, Nadine El Kassis, Laura-Maria Maalouly, Malak Moubarak, Mary Dib, Michel Ghossain

**Affiliations:** ^1^Department of Radiology, Hôtel-Dieu de France University Hospital, Saint Joseph University, Beirut, Lebanon; ^2^Department of Gynecology and Obstetrics, Hôtel-Dieu de France University Hospital, Saint Joseph University, Beirut, Lebanon; ^3^Department of Medical Oncology, Hôtel-Dieu de France University Hospital, Saint Joseph University, Beirut, Lebanon; ^4^Faculty of Pharmacy, Lebanese American University, Byblos, Lebanon; ^5^Department of Radiology, University Medical Center-Rizk Hospital, Beirut, Lebanon

## Abstract

**Purpose:**

To study the distribution of breast mammogram density in Lebanese women and correlate it with breast cancer (BC) incidence.

**Methods:**

Data from 1,049 women who had screening or diagnostic mammography were retrospectively reviewed. Age, menopausal status, contraceptives or hormonal replacement therapy (HRT), parity, breastfeeding, history of BC, breast mammogram density, and final BI-RADS assessment were collected. Breast density was analyzed in each age category and compared according to factors that could influence breast density and BC incidence.

**Results:**

120 (11.4%) patients had BC personal history with radiation and/or chemotherapy; 66 patients were postmenopausal under HRT. Mean age was 52.58 ± 11.90 years. 76.4% of the patients (30–39 years) had dense breasts. Parity, age, and menopausal status were correlated to breast density whereas breastfeeding and personal/family history of BC and HRT were not. In multivariate analysis, it was shown that the risk of breast cancer significantly increases 3.3% with age (*P* = 0.005), 2.5 times in case of menopause (*P* = 0.004), and 1.4 times when breast density increases (*P* = 0.014).

**Conclusion:**

Breast density distribution in Lebanon is similar to the western society. Similarly to other studies, it was shown that high breast density was statistically related to breast cancer, especially in older and menopausal women.

## 1. Introduction

Breast cancer (BC) is the most common malignancy in women worldwide, with nearly 1.7 million new cases diagnosed in 2012. The highest age-standardized incidence rates are reported in the developed countries, ranging from 111.9 in Belgium to 92.9 in USA and 83.1 per 100,000 habitants in Switzerland [1]. In parallel, Africa, South America, and Eastern, Southeastern, and Western Asia have lower incidence [1–3].

In Lebanon, a Middle Eastern Asian country with a population size of 4 million people, BC is the most frequent cancer and constitutes 38% of all women cancers [4, 5]. The age-standardized incidence rate of BC is 76.15 per 100,000 persons, thus lower than the western countries but higher than the surrounding countries such as Mediterranean Europe, Eastern Europe, and Arab countries [2, 6]. Most importantly, the median age (52 years) of BC in Lebanon is lower than the western population (63 years) but higher than the Arab countries (e.g., 46 years in Egypt) [6–8]. Nonetheless, Lebanese age-specific incidence rate is the highest worldwide for the 35–39 and 40–49 age groups with the exception of Israeli Jews in the 35–39 age group [9, 10].

Several papers have found that high breast density, besides decreasing mammography sensitivity by masking lesions, is a strong independent risk factor of BC [11–14]. Breast density is also partially genetically inherited [15–17], and there is increasing interest in the idea that the genetic variants may be responsible for BC subtypes [18]. Moreover, cancers in extremely dense breasts occur in younger women and appear to be phenotypically different from those arising in other breast density groups [18].

Since Lebanese age-specific incidence rate is the highest worldwide for the 35–49 age group, we aimed to study the distribution of breast density in the Lebanese population and to compare it with the western population. We also tested the association between breast cancer and breast density in Lebanon.

## 2. Materials and Methods

### 2.1. Study Design

This was an observational retrospective study. It was conducted from September 2010 till March 2012 in two university hospitals in Lebanon. Lebanese women who underwent mammography exam (screen film mammography or digital mammography [GE, GE Health Care, USA)]) were eligible for inclusion. Women with bilateral BC or under chemotherapy were excluded.

### 2.2. Data Collection

Data were collected on a sheet before the mammogram exam as part of the routine inquiry. The sheet included information on the purpose of the exam (screening or clinical abnormality such as pain, palpable lump, nipple discharge or retraction, and skin change), patient age, age at menarche, postmenopausal or premenopausal status, hormonal replacement therapy (HRT) or contraceptive uptake, parity, personal or familial history of breast or ovarian cancer, age of onset of personal or familial BC, and gynecological personal history. After the mammogram was done, breast density was assessed visually on the two standard views (craniocaudal and mediolateral oblique) for each side by one reader and noted on the inquiry sheet. Breast density was evaluated on hard copies and computer screen.

Breast density was classified according to BI-RADS version 4 [22] as type I (<25%, almost entirely fatty), type II (25–50%, scattered fibroglandular densities), type III (51–75% glandular, heterogeneously dense), and type IV (>75% glandular, extremely dense breast [22]). The mammogram assessment upon the recommendation of the American College of Radiology (ACR) was noted at the end of the exam, for each breast side, that is, BI-RADS 0 = possible finding, need for additional imaging information, BI-RADS 1 = no abnormality is found, BI-RADS 2 = benign findings, BI-RADS 3 = probably benign abnormality (risk of malignancy < 2%), BI-RADS 4 = biopsy is required (risk of malignancy between 2 and 94%), BI-RADS 5 = highly suggestive of malignancy (risk of malignancy > 94%), and BI-RADS 6 = known biopsy-proven malignancy [22]. In case of personal history of BC, breast density was evaluated in the noncancerous breast. Familial risk was classified as none, minor, and moderate in case of, respectively, no case, one history, two histories of breast or ovarian cancer and high in case of three histories of breast or ovarian cancer in the same familial branch, of bilateral BC or male BC. Asymmetry in breast density was rarely encountered and the score of the denser breast was retained.

### 2.3. Statistical Analysis

Patients were divided into six age groups (<30, 30–39, 40–49, 50–59, 60–69, and >70 years old). When only two categories of breast density were needed, types I and II according to BI-RADS were classified as nondense breast and type III and VI according to BI-RADS as dense breast. The patients' characteristics were summarized as mean and standard deviation for continuous variables and frequencies [*n*(%)] for categorical variables. Comparisons between patients with and without breast cancer were compared using Chi-square (*χ*^2^) and Fisher's exact tests as appropriate, for clinical characteristics. Also, comparisons between the four categories of breast density were performed using ANOVA, *χ*^2^, and Fisher's exact tests. Sensitivity analysis was done to test the relationship between breast density and breast cancer by clinical characteristics. Finally, a multivariate analysis was performed to test the association between breast cancer (outcome) and breast density (independent variable), having age, menopause, HRT, parity, breastfeeding, and history of breast cancer as covariates. Statistical significance was two-sided and set at the 5%. The statistical analysis was performed using IBM SPSS version 21 for windows release (IBM Corp. Released 2012. IBM SPSS Statistics for Windows, Version 21.0. Armonk, NY: IBM Corp.).

### 2.4. Ethical Considerations

This study was approved by the ethics committees (ECs) of the participating centers. Verbal consent was obtained over the phone from the participants. The ECs considered our observational study as a less than minimal risk research study since there were no known physical, emotional, psychological, or economical risk and no special populations (i.e., minors, prisoners, and pregnant women) were involved. It required no specific consultation and there was no administration of any investigational product. This study did not involve any change in the clinical management of the patients. All data were treated in respect to the patients' anonymity and confidentiality.

## 3. Results

### 3.1. Patients' Characteristics

Overall, 1,049 eligible patients were included of whom 955 (91%) patients had screening mammogram and 94 (9%) a diagnostic mammogram for variable reasons (pain, palpable mass, or nipple discharge). The mean age of the patients was 52.58 ± 11.90 years. In total, 33.5% of the patients were aged between 40 and 49 years and 27.8% between 50 and 59 years. The mean parity was 2.29 ± 1.60, and 546 women had breastfed (56.5%). Overall, 90.9% of patients had no hormonal uptake (HRT or contraceptives), and 553 (53.1%) patients were postmenopausal of whom 11.9% were under HRT. Also, 46.9% were premenopausal, of whom 5.9% were under hormonal contraception (Table 1).

One hundred and twenty patients had personal history of BC (11.4%). The mean age of the patients at the onset of their BC was 43 ± 21.69 years. Considering the risk of having BC, 72.2% of the patients had no risk factors, 11.9% had a low risk, 11.6% had a moderate risk, and 4.3% had high risk factors. In total 95% of the mammograms were classified as BI-RADS 1 or 2 (normal or benign findings).

### 3.2. Distribution of Breast Density in Lebanese Population

**Figure 1:**
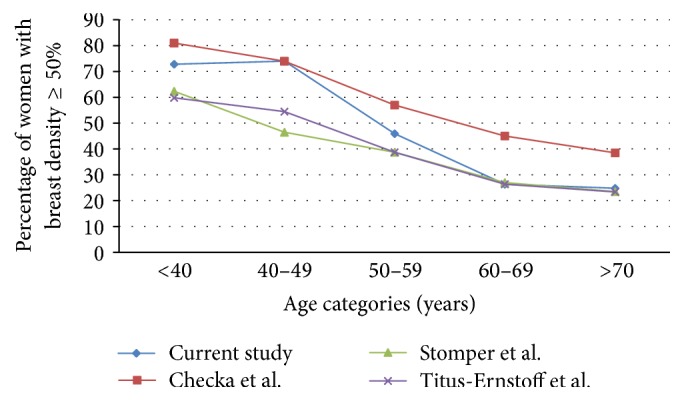
Comparison in the percentage of females with breast density of 50% and above between our study's results and the studies of Stomper et al. [20], Titus-Ernstoff et al. [21], and Checka et al. [19].

102/1,049 (9.7%) women had type I breast density, 393 (37.5%) had type II breast density, 481 (45.9%) had type III breast density, and 73 (7%) had type IV breast density. Almost half of the women (53%) had dense breasts. Also, breast density decreased with age (Tables 2(a) and 2(b) and Figure 1).

### 3.3. Comparison between the Distribution of Breast Density in the Lebanese Population and the Western Population

The comparison of the distribution of women by age category between our sample and the findings of the three studies by Stomper et al. in 1,353 women [20], Titus-Ernstoff et al. (*n* = 133,772) [21], and Checka et al. (*n* = 6595) [19] are displayed in Table 3. All studies revealed a parallelism in the distribution of breast density, with breast density being higher in younger ages and lower in the older groups. For women aged 40–50 years and younger, breast density in Lebanese population was higher than reported by Stomper et al. [20] and Titus-Ernstoff et al. [21] but slightly similar to the results by Checka et al. [19] (Figure 1).

### 3.4. Association between Breast Cancer, Breast Density, and Other Risk Factors

**Figure 2:**
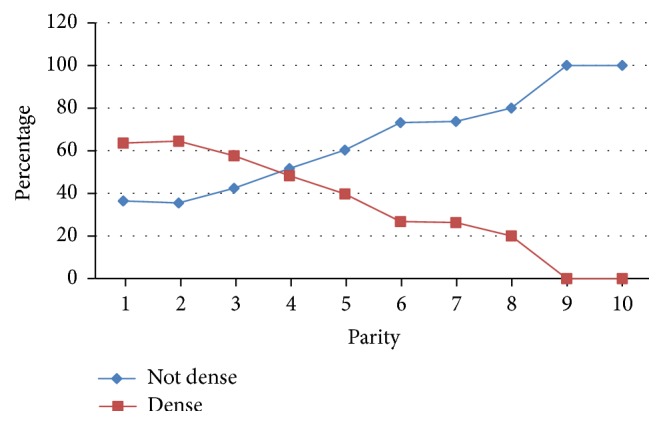
Parity with respect to breast density levels.

The hormonal status of women (postmenopausal or premenopausal) and the number of living children, with a cut-off starting with 1 child, were statistically related to breast density (*P* < 0.001 for both variables; ANOVA and Fisher tests) (Tables 2(b) and 4 and Figure 2). Among 553 postmenopausal women, 190 had dense breast (34.4%) and 363 had nondense breasts (65.6%). Also, 360 out of 488 premenopausal women had dense breast (73.8%) and 128 had nondense breasts (26.2%). However, HRT, breastfeeding, and personal or family history of BC were not statistically related to breast density (Table 4). Finally, 37.5% of the patients with BI-RADS 0 had dense breasts. No statistical differences between the four categories of breast density were observed for the breast final report classification, whatever the combination used (Table 4), that is, final report assessments categories 0 versus 1, 2, 3, 4, and 5; categories 0, 3, 4, and 5 versus 1 and 2 (BI-RADS screening type statistics); categories 4 and 5 versus 1, 2, and 3 (BI-RADS diagnostic type statistics) [22]. The most appropriate combination in our study is the diagnostic type statistics, as defined in the BI-RADS [22], because, in our practice, when a patient comes for a screening study, the latter is immediately completed by a diagnostic study if necessary, and only one report is issued; this statistic excludes category 0 (*n* = 8 patients) and category 6 (*n* = 2 patients) corresponding to a trivial number compared to the total of 1,049 patients.

In univariate analysis, breast density and breast cancer were significantly correlated in patients aged more than 70 years (*P* = 0.004) and in menopausal women (*P* = 0.017). No significant correlation was detected between breast density and breast cancer, by HRT, parity, and type of mammogram. In multivariate analysis, it was shown that the risk of breast cancer significantly increases 3.3% with age (beta coefficient = 0.347, exp beta = 1.033, and *P* = 0.005), 2.5 times in case of menopause (beta coefficient = 0.879, exp beta = 2.409, and *P* = 0.004), and 1.4 times when breast density increases (beta coefficient = 0.347, exp beta = 1.415, and *P* = 0.014).

## 4. Discussion

This is the first study evaluating the relation between mammographic breast density and BC risk factors in Lebanon and Middle East countries (from Turkey to Egypt including Iran and Arab peninsula). First, it reveals that the distribution of Lebanese women with dense breast is similar to those of international studies [19–21], we had the same breast density distribution particularly in the extreme age groups, about 30% of patients under 30 years old have fatty breasts, and about 25% over 70 years old have dense breasts. Importantly, a study by del Carmen et al. showed that mammographic breast density does not differ across ethnicities, which allows comparing our results with the international data [23]. Second, the inverse relationship between breast density and patient age agrees with most of the existing literature in this area [18, 21, 24]. High breast density was predominant in groups under 50 years old, while trend toward a lower breast density was seen in older women.

Our analyses also provide important new information about breast density and its risk factors in Lebanese women. We found that parity and menopausal status were associated with breast density changes whereas breastfeeding and personal and family history of BC and HRT were not. Parity was related to lower breast density with a cut-off of 4 children, this number being higher than the cut-off of 2 children as reported by Stomper et al. [20]. As suggested by correlations observed in this study and other studies [25, 26], some of the changes in breast composition induced by parity are reflected by a reduced mammographic density. An explanation is that parity leads to changes in breast morphology (e.g., number and differentiation status of the lobular structures [27]), histology (e.g., amount of collagen [28]), and biochemistry (e.g., gene expression patterns [29, 30]). Moreover, dense tissue has generally been associated with younger age and premenopausal status, with the assumption that breast density gradually decreases after menopause. These data were confirmed in our study with a *P* < 0.001 (ANOVA test) (Table 2(b)).

Contrary to the database of 35,019 postmenopausal women enrolled in a population-based mammography-screening program in the USA [31], our analysis revealed that having a family or personal history of BC in Lebanon did not influence mammographic breast density. Other studies have also clearly shown mammographic density as a heritable risk factor [32, 33]. In particular, twin studies have shown that percent mammographic density, at a given age, is highly heritable, with 60% of the variation in breast density being explained by genetic factors [15, 34]. Also, HRT that was identified as an environmental factor affecting breast density [35] was not associated with breast density changes in our study population as many studies did [36].

In our series, 13 women under 30 years old and 110 women between 30 and 39 years old had mammography (Table 1). This relatively large number is due to the fact that part of these women has a cancer, a palpable mass, or a family history of cancer necessitating mammography. Another reason not to be neglected is the extreme anxiety generated in friends and neighbors of a young woman (<30 or <40 years old) that had a BC especially in the absence of a family history. Another reason was women preferences. Although the risk of mammographic examination at young age was explained by the clinicians, some women were persistent about being imaged with mammography.

Furthermore, our study reported that BC was significantly associated with breast density, as the risk of BC appeared to be increasing with breast density, especially in elder menopausal women. Our findings are consistent with the results of a meta-analysis of 42 publications on the association between breast density and BC [37]. While Warren identified HRT as a risk factor that modifies the association between BC and breast density [38], HRT use did not modify BC risk in our study, which is consistent with two previous studies [39, 40]. In our study, family history of BC did not increase the association between BC and breast density, contrary to two American studies which concluded that BC risk may be associated with genetic factors that determine breast density [21, 41]. Additionally, our study found a positive association between BC and breast density in older and menopausal women. Nonetheless, a common consensus was not reached in this sense as literature shows controversial results: some studies suggested a positive association in postmenopausal women [39, 42, 43], whereas other studies found a negative or no association in these women [44, 45].

### 4.1. Study Strengths

BI-RADS version 5 for the classification of breast mammogram density was not used since it was published after the beginning of the study, which therefore would not be comparable to previous studies. Moreover, version 5 is less suited for this study because a breast with less than 50% of glandular tissue is classified C if a small region is of high density (A, B, C, and D replaced I, II, II, and IV) [46]. The model of this study could be used for assessing populations of other countries or regions.

### 4.2. Study Limitations

Several limitations can be pointed out. While the number of women in this study is not negligible, close to that of Stomper et al. [20], a larger number would give more reliable statistics. Combining materials with more than two university hospitals in this country or neighboring countries would lead to an interesting study. Also, a bias may be introduced by the fact that the 1,049 women included in this study do not reflect accurately the entire Lebanese populations.

## 5. Conclusion

To conclude, breast density distribution in Lebanon was similar to the western society. Moreover, our study showed breast density to be a significant risk factor for BC, especially in older and menopausal women, similarly to literature findings.

## Figures and Tables

**Table 1:** Sociodemographic characteristics of the 1,049 Lebanese women.

	No breast cancer		Breast cancer		*P* value	All patients	
	*n*	%	*n*	%		*n*	%
Age (years)	*N* = 929 51.63 ± 11.70 (range: 18–89)		*N* = 120 58.75 ± 11.65 (range: 32–83)		<10^−3*∗*^	*N* = 1,049 52.58 ± 11.90 (range: 18–89)	
<30	13	1.4%	0	0.0%		13	1.2%
30–39	106	11.4%	4	3.3%		110	10.5%
40–49	325	35.0%	26	21.7%		351	33.5%
50–59	252	27.1%	40	33.3%		292	27.8%
60–69	148	15.9%	26	21.7%		174	16.6%
>70	85	9.1%	24	20.0%		109	10.4%

Menopause	*N* = 921		*N* = 120		<10^−3*∗*^	*N* = 1,041	
Yes	460	49.9%	93	77.5%		553	53.1%
No	461	50.1%	27	22.5%		488	46.9%

Hormone replacement therapy or hormone contraception	*N* = 929		*N* = 120		<10^−3*∗*^	*N* = 1,049	
Yes	95	10.2%	0	0%		95	9.1%
No	834	89.8%	120	100%		954	90.9%

Type of mammogram	*N* = 929		*N* = 120		<10^−3*∗*^	*N* = 1,049	
Screening	886	95.4%	69	57.5%		955	91.0%
Diagnostic	43	4.6%	51	42.5%		94	9.0%

Breastfeeding	*N* = 859		*N* = 104			*N* = 967	
Yes	377	43.9%	40	38.5%		546	56.5%
No	482	56.1%	64	61.5%		421	43.5%

Parity	*N* = 862 2.26 ± 1.60 (range: 0–9)		*N* = 159 2.55 ± 1.66 (range: 0–8)		0.479	*N* = 973 2.29 ± 1.60 (range: 0–9)	
0	176	20.4%	19	11.9%		195	20.0%
1	70	8.1%	6	3.8%		76	7.8%
2	233	27.0%	22	13.8%		255	26.2%
3	223	25.9%	38	23.9%		261	26.8%
4	102	11.8%	14	8.8%		116	11.9%
5	36	4.2%	55	34.6%		41	4.2%
6	16	1.9%	3	1.9%		19	2.0%
7	4	0.5%	1	0.6%		5	0.5%
8	2	0.2%	1	0.6%		3	0.3%
9	2	0.2%	0	0.0%		2	0.2%

^*∗*^Significance level set at 5%.

**Table:** (a) Distribution of breast density in Lebanese women (*n* = 1,049).

Age (years)	Breast density category							
	I		II		III		IV	
	*n*	%	*n*	%	*n*	%	*n*	%
<30	0	0.0%	4	30.8%	4	30.8%	5	38.5%
30–39	4	3.6%	22	20.0%	72	65.5%	12	10.9%
40–49	8	2.3%	87	24.8%	216	61.5%	40	11.4%
50–59	30	10.3%	128	43.8%	124	42.5%	10	3.4%
60–69	33	19.0%	97	55.7%	41	23.6%	3	1.7%
>70	27	24.8%	55	50.5%	24	22.0%	3	2.8%

*Total*	*102*	*9.7%*	*393*	*37.5%*	*481*	*45.9%*	*73*	*7.0%*

**Table:** (b) Mean age and parity in different breast density groups (*n* = 1,049).

	Breast density	*N*	Mean	Standard deviation	Minimum	Maximum	*P* value^*∗*^	
Age	I	102	62.18	11.43	33.00	84.00	<0.001	
	II	393	56.41	11.32	28.00	82.00		
	III	481	48.52	10.16	18.00	89.00		
	IV	73	45.34	10.96	25.00	82.00		
	Total	1,049	52.59	11.90	18.00	89.00		

Parity	I	99	2.93	1.85	0.00	8.00	<0.001	
	II	365	2.58	1.64	0.00	9.00		
	III	447	2.00	1.44	0.00	7.00		
	IV	62	1.66	1.44	0.00	6.00		
	Total	973	2.29	1.60	0.00	9.00	

^*∗*^Significance level set at 5%. ANOVA test shows that the breast density decreased when age (*P* value < 0.001) and parity (*P* value < 0.001) increase.

**Table 3:** Proportion of females in each age category for our study and the previous studies of Checka et al., Stomper et al., and Titus-Ernstoff et al.

Age category (years)	Current study		Checka et al. [19]		Stomper et al. [20]		Titus-Ernstoff et al. [21]	
	*n*	%	*n*	%	*n*	%	*n*	%
<40	123	11.7%	249	3.8%	351	26.0%	16,739	12.5%
40–49	351	33.5%	1,675	25.5%	250	18.5%	38,384	28.7%
50–59	292	27.8%	2,192	33.2%	251	18.5%	35,134	26.3%
60–69	174	16.6%	1,639	24.8%	251	18.5%	30,127	22.5%
70–79	109	10.4%	840	12.7%	250	18.5%	133,88	10.0%

*Total*	*1,049*	*100%*	*6,595*	*100%*	*1,353*	*100%*	*133,772*	*100%*

**Table 4:** Comparison between the four categories of breast density by clinical characteristics (*n* = 1,049).

	Breast density category								*P* value test^*∗*^
	I		II		III		IV		
	*n*	%	*n*	%	*n*	%	*n*	%	
*Menopause*									*Fisher*: <0.001
Yes	90	16.3%	273	49.4%	171	30.9%	19	3.4%	
No	11	2.3%	117	24.0%	306	62.7%	54	11.1%	
Total	101	9.7%	390	37.5%	477	45.8%	73	7.0%	

*Breastfeeding*									*χ* ^2^: 0.181, *Fisher*: 0.176
Yes	57	10.4%	221	40.5%	236	43.2%	32	5.9%	
No	41	9.7%	144	34.2%	206	48.9%	30	7.1%	
Total	102	10.1%	393	37.7%	481	45.7%	73	6.4%	

*Parity*									*Fisher*: <0.001
0	15	7.7%	56	28.7%	103	52.8%	21	10.8%	
1	4	5.3%	23	30.3%	45	59.2%	4	5.3%	
2	16	6.3%	92	36.1%	128	50.2%	19	7.5%	
3	32	12.3%	103	39.5%	113	43.3%	13	5.0%	
4	17	14.7%	53	45.7%	42	36.2%	4	3.4%	
5	6	14.6%	24	58.5%	11	26.8%	0	0.0%	
6	5	26.3%	9	47.4%	4	21.1%	1	5.3%	
7	2	40.0%	2	40.0%	1	20.0%	0	0.0%	
8	2	66.7%	1	33.3%	0	0.0%	0	0.0%	
9	0	0.0%	2	100.0%	0	0.0%	0	0.0%	
Total	99	10.2%	365	37.5%	447	45.9%	62	6.4%	

*Parity cut-off*									*Fisher*: <0.001
≤1	19	7.0%	79	29.2%	148	54.6%	25	9.2%	
>1	80	11.4%	286	40.7%	299	42.6%	37	5.3%	

*HRT in postmenopausal women*									*Fisher*: 0.829
No	80	16.4%	241	49.5%	148	30.4%	18	3.7%	
Yes	10	15.2%	32	48.5%	23	34.8%	1	1.5%	

*Breast cancer*									*Fisher*: 0.567
Yes	15	12.5%	44	36.7%	51	42.5%	10	8.3%	
No	87	9.4%	349	37.6%	430	46.3%	63	6.8%	
Total	102	9.7%	393	37.5%	481	45.9%	73	7.0%	

*Family history of breast cancer*									*Fisher*: 0.124
Yes	25	8.6%	105	36.0%	133	45.5%	29	9.9%	
No	77	10.2%	288	38.0%	348	46.0%	44	5.8%	
Total	102	9.7%	393	37.5%	481	45.9%	73	7.0%	

*Risk assessment of breast cancer*									*Fisher*: 0.293
No	77	10.2%	288	38.0%	348	46.0%	44	5.8%	
Low	11	8.8%	44	35.2%	55	44.0%	15	12.0%	
Moderate	13	10.7%	44	36.1%	56	45.9%	9	7.4%	
High	1	2.2%	17	37.8%	22	48.9%	5	11.1%	
Total	102	9.7%	393	37.5%	481	45.9%	73	7.0%	

*BI-RADS final report classification *									*Fisher*: 0.056
0	0	0.0%	3	37.5%	3	37.5%	2	25.0%	
1	22	17.5%	47	37.3%	54	42.9%	3	2.4%	
2	68	9.6%	261	36.7%	335	47.1%	47	6.6%	
3	9	5.6%	64	40.0%	69	43.1%	18	11.3%	
4	2	5.9%	14	41.2%	16	47.1%	2	5.9%	
5	1	12.5%	3	37.5%	3	37.5%	1	12.5%	
6	0	0.0%	1	50.0%	1	50.0%	0	0.0%	
Total	102	9.7%	393	37.5%	481	45.9%	73	7.0%	

*Screening type statistics*	No dense breast (*n*, %)				Dense breast (*n*, %)				*χ* ^2^: 0.634
Categories 0, 3, 4, 5	96		45.7%		114		54.3%		
Categories 1, 2	398		47.6%		439		52.4%		

*Diagnostic type statistics*	No dense breast (*n*, %)				Dense breast (*n*, %)				*Fisher*: 0.962
Categories 4, 5	20		47.6%		22		52.4%		
Categories 1, 2, 3	471		47.2%		526		52.8%		

*0 versus 1, 2, 3, 4, 5*	No dense breast (*n*, %)				Dense breast (*n*, %)				*Fisher*: 0.729
Categories 0	3		37.5%		5		62.5%		
Categories 1, 2, 3, 4, 5	491		47.3%		548		52.7%		

^*∗*^Significance level set at 5%.

## References

[1] World Cancer Research Fund International Breast cancer statistics. http://www.wcrf.org/int/cancer-facts-figures/data-specific-cancers/breast-cancer-statistics.

[2] Ferlay J., Shin H. R., Bray F., Forman D., Mathers C., Parkin D. M. (2010). Estimates of worldwide burden of cancer in 2008: GLOBOCAN 2008. *International Journal of Cancer*.

[3] Jemal A., Bray F., Center M. M., Ferlay J., Ward E., Forman D. (2011). Global cancer statistics. *CA: A Cancer Journal for Clinicians*.

[4] Shamseddine A., Sibai A.-M., Gehchan N. (2004). Cancer incidence in postwar Lebanon: Findings from the first national population-based registry, 1998. *Annals of Epidemiology*.

[5] 2003 National Cancer Registry, Lebanon, February, 2006. http://www.moph.gov.lb/Publications/Documents/NCR2003.pdf.

[6] El Saghir N. S., Shamseddine A. I., Geara F. (2002). Age distribution of breast cancer in Lebanon: Increased percentages and age adjusted incidence rates of younger-aged groups at presentation. *Journal Medical Libanais*.

[7] Ries L. A. G., Melbert D., Krapcho M. SEER Cancer Statistics Review, 1975-2005, National Cancer Institute. http://seer.cancer.gov/csr/1975_2005/.

[8] Najjar H., Easson A. (2010). Age at diagnosis of breast cancer in Arab nations. *International Journal of Surgery*.

[9] Denewer A., Hussein O., Farouk O., Elnahas W., Khater A., El-Saed A. (2010). Cost-effectiveness of clinical breast assessment-based screening in rural Egypt. *World Journal of Surgery*.

[10] Lakkis N. A., Adib S. M., Osman M. H., Musharafieh U. M., Hamadeh G. N. (2010). Breast cancer in Lebanon: Incidence and comparison to regional and Western countries. *Cancer Epidemiology*.

[11] Brisson J., Diorio C., Mâsse B. (2003). Wolfe's parenchymal pattern and percentage of the breast with mammographic densities: redundant or complementary classifications?. *Cancer Epidemiology Biomarkers and Prevention*.

[12] Martin L. J., Melnichouk O., Guo H. (2010). Family history, mammographic density, and risk of breast cancer. *Cancer Epidemiology Biomarkers and Prevention*.

[13] Bertrand K. A., Scott C. G., Tamimi R. M. (2015). Dense and nondense Mammographic area and risk of breast cancer by age and tumor characteristics. *Cancer Epidemiology Biomarkers and Prevention*.

[14] Harvey J. A., Gard C. C., Miglioretti D. L. (2013). Reported mammographic density: Film-screen versus digital acquisition. *Radiology*.

[15] Ursin G., Lillie E. O., Lee E. (2009). The relative importance of genetics and environment on mammographic density. *Cancer Epidemiology Biomarkers and Prevention*.

[16] Lee E., Haiman C. A., Ma H., Van Den Berg D., Bernstein L., Ursin G. (2008). The role of established breast cancer susceptibility loci in mammographic density in young women. *Cancer Epidemiology Biomarkers and Prevention*.

[17] Tamimi R. M., Cox D., Kraft P., Colditz G. A., Hankinson S. E., Hunter D. J. (2008). Breast cancer susceptibility loci and mammographic density. *Breast Cancer Research*.

[18] Arora N., King T. A., Jacks L. M. (2010). Impact of breast density on the presenting features of malignancy. *Annals of Surgical Oncology*.

[19] Checka C. M., Chun J. E., Schnabel F. R., Lee J., Toth H. (2012). The relationship of mammographic density and age: implications for breast cancer screening. *The American Journal of Roentgenology*.

[20] Stomper P. C., D'Souza D. J., DiNitto P. A., Arredondo M. A. (1996). Analysis of parenchymal density on mammograms in 1353 women 25-79 years old. *American Journal of Roentgenology*.

[21] Titus-Ernstoff L., Tosteson A. N. A., Kasales C. (2006). Breast cancer risk factors in relation to breast density (United States). *Cancer Causes and Control*.

[22] Vanel D. (2007). The American College of Radiology (ACR) Breast Imaging and Reporting Data System (BI-RADS™): A step towards a universal radiological language?. *European Journal of Radiology*.

[23] Del Carmen M. G., Halpern E. F., Kopans D. B. (2007). Mammographic breast density and race. *American Journal of Roentgenology*.

[24] Liu J., Liu P.-F., Li J.-N. (2014). Analysis of mammographic breast density in a group of screening Chinese women and breast cancer patients. *Asian Pacific Journal of Cancer Prevention*.

[25] Tamburrini A.-L., Woolcott C. G., Boyd N. F. (2011). Associations between mammographic density and serum and dietary cholesterol. *Breast Cancer Research and Treatment*.

[26] Woolcott C. G., Koga K., Conroy S. M. (2012). Mammographic density, parity and age at first birth, and risk of breast cancer: an analysis of four case-control studies. *Breast Cancer Research and Treatment*.

[27] Russo J., Hu Y., Yang X., Russo I. H. (2000). Chapter 1: Developmental, Cellular, and Molecular Basis of Human Breast Cancer. *JNCI Monographs*.

[28] Li T., Sun L., Miller N. (2005). The association of measured breast tissue characteristics with mammographic density and other risk factors for breast cancer. *Cancer Epidemiology Biomarkers and Prevention*.

[29] Balogh G., Heulings R., Mailo D. (2006). Genomic signature induced by pregnancy in the human breast. *International Journal of Oncology*.

[30] Medina D. (2005). Mammary developmental fate and breast cancer risk. *Endocrine-Related Cancer*.

[31] Crest A. B., Aiello E. J., Anderson M. L., Buist D. S. M. (2006). Varying levels of family history of breast cancer in relation to mammographic breast density (United States). *Cancer Causes and Control*.

[32] Boyd N. F., Martin L. J., Rommens J. M. (2009). Mammographic density: A heritable risk factor for breast cancer. *Methods in Molecular Biology*.

[33] Vachon C. M., Sellers T. A., Carlson E. E. (2007). Strong evidence of a genetic determinant for mammographic density, a major risk factor for breast cancer. *Cancer Research*.

[34] Sung J., Song Y.-M., Stone J., Lee K., Jeong J.-I., Kim S.-S. (2010). Genetic influences on mammographic density in Korean twin and family: The healthy twin study. *Breast Cancer Research and Treatment*.

[35] Rutter C. M., Mandelson M. T., Laya M. B., Taplin S., Seger (2001). Changes in breast density associated with initiation, discontinuation, and continuing use of hormone replacement therapy. *Journal of the American Medical Association*.

[36] Tesic V., Kolaric B., Znaor A., Kuna S. K., Brkljacic B. (2013). Mammographic density and estimation of breast cancer risk in intermediate risk population. *Breast Journal*.

[37] McCormack V. A., dos Santos Silva I. (2006). Breast density and parenchymal patterns as markers of breast cancer risk: a meta-analysis. *Cancer Epidemiology Biomarkers & Prevention*.

[38] Warren R. (2004). Hormones and mammographic breast density. *Maturitas*.

[39] Lam P. B., Vacek P. M., Geller B. M., Muss H. B. (2000). The association of increased weight, body mass index, and tissue density with the risk of breast carcinoma in Vermont. *Cancer*.

[40] Duffy S. W., Jakes R. W., Ng F. C., Gao F. (2004). Interaction of dense breast patterns with other breast cancer risk factors in a case-control study. *British Journal of Cancer*.

[41] Ziv E., Shepherd J., Smith-Bindman R., Kerlikowske K. (2003). Mammographic breast density and family history of breast cancer. *Journal of the National Cancer Institute*.

[42] Ursin G., Ma H., Wu A. H. (2003). Mammographic density and breast cancer in three ethnic groups. *Cancer Epidemiology Biomarkers and Prevention*.

[43] Park I. H., Ko K., Joo J. (2014). High Volumetric Breast Density Predicts Risk for Breast Cancer in Postmenopausal, but not Premenopausal, Korean Women. *Annals of Surgical Oncology*.

[44] Boyd N. F., Guo H., Martin L. J. (2007). Mammographic density and the risk and detection of breast cancer. *The New England Journal of Medicine*.

[45] Kim B.-K., Choi Y.-H., Nguyen T. L. (2015). Mammographic density and risk of breast cancer in Korean women. *European Journal of Cancer Prevention*.

[46] (January 2014). *ACR BI-RADS Mammography Atlas*.

